# Weekday–Weekend Differences in Chrononutritional Variables Depend on Urban or Rural Living

**DOI:** 10.3390/nu17010108

**Published:** 2024-12-30

**Authors:** Jefferson Souza Santos, Cibele Aparecida Crispim, Debra Jean Skene, Claudia Roberta de Castro Moreno

**Affiliations:** 1 Department of Health and Society, School of Public Health, University of São Paulo, São Paulo 01246-904, Brazil; jeffersonsouza@ufpr.br; 2 Department of Theory and Foundations of Education, Education Sector, Federal University of Paraná, Curitiba 80230-130, Brazil; 3 Graduate Program in Health Sciences, Faculty of Medicine, Federal University of Uberlândia, Uberlândia 38405-320, Brazil; cibele.crispim@ufu.br; 4 Chronobiology Section, Faculty of Health and Medical Sciences, University of Surrey, Guildford GU2 7XH, UK; d.skene@surrey.ac.uk

**Keywords:** chrononutrition, meal time, urbanization, circadian misalignment, family budget survey

## Abstract

Background/Objectives: Studies have highlighted the impact of work and school schedules on food preferences, suggesting that individuals’ dietary choices may change during the week to align with their daily routines. Despite the variation in food composition in the population, there is no evidence identifying differences in food intake times and composition across the days of the week in urban/rural locations. Thus, the study’s aim was to identify weekday vs. weekend differences in food intake times and composition (calories) between urban and rural areas. Methods: Data from 5770 participants (aged 18–59 years) were analyzed from the National Household Budget Survey (POF-IBGE) consisting of two distinct food diary records (weekday + weekend) per individual, including area (urban or rural) information in Brazil. Results: During weekdays, the time of the first food intake was significantly earlier, and the last food intake time was significantly later compared to weekends, resulting in a longer eating window on weekdays in both urban and rural areas. People living in urban areas exhibited delayed first and last food intake times, resulting in later caloric and eating midpoints compared to people living in rural areas. Periodogram analysis detected weekly rhythmicity (7 days) at the time of the first food intake and the length of the eating window in urban residents. Conclusions: The observed 7-day rhythmic pattern of food intake in urban areas, driven by work and school schedules, underscores the influence of urbanization on dietary timing and composition. In contrast, rural areas exhibited more stable and earlier eating patterns. These results emphasize the need for public health interventions to address meal timing and circadian alignment, particularly in urban settings, to mitigate the risk of metabolic disorders and improve overall health outcomes.

## 1. Introduction

The timing of meals (chrononutrition) and its metabolic consequences have received enhanced research interest in recent years [1,2]. Knowledge about “when we eat” combined with the substantial literature about “what we eat” across the 24 h day has contributed to understanding the factors driving overweight and obesity in the population [3,4,5]. Evidence from chrononutrition studies has revealed the potential of unhealthy dietary habits to influence circadian rhythms and metabolic health involving bidirectionality between the main circadian pacemaker in the brain (located in the hypothalamic suprachiasmatic nuclei, SCN) and the peripheral clocks located in organs involved in food intake and metabolism [3,6,7]. Studies have shown that the timing of food intake has the potential to impact nutritional and metabolic health such as body weight [8,9], hormonal balance [10,11], glycemic and lipid metabolism [12], and sleep quality [13,14,15].

Weekend–weekday differences in the timing of food depend on rural/urban living. Variation in food intake times has been associated with a range of metabolic outcomes, including both positive and negative health effects [16,17]. Indeed, weight gain and obesity have been associated with breakfast-skipping behavior [1,8,18]. Eating breakfast has been associated with a healthy metabolic status including positive outcomes after breakfast intake on glucose, insulin, ghrelin, and hunger [1,19,20]. Furthermore, a high-calorie breakfast together with an opposite low-calorie dinner seems to be effective in treating overweight/obesity and other metabolic disturbances [3]. Other recent studies have found that eating late is also a behavior associated with weight gain and obesity, suggesting a relationship between eating late and higher energy intake from total fats, cholesterol, and carbohydrates [1,19]. During the nighttime, fat oxidation is compromised and overall metabolism becomes less efficient due to reduced glucose tolerance [1]. In addition, societal routines and pressures such as work/school demands have explained the late eating behavior leading people to prefer unhealthy food [21]. Therefore, not only does the quality and quantity of meals exert a crucial role in health but also food intake times need to be considered.

The Dietary Guidelines for the Brazilian Population recommend consuming fresh or minimally processed foods, cooking meals at home, eating in appropriate environments, avoiding ultra-processed products, and maintaining regular meal times with three main meals (breakfast, lunch, and dinner) to promote health and well-being [22]. A recent study by Rodrigues et al. [23], analyzing representative data from the Brazilian population (46,164 individuals aged ≥10 years), showed that the vast majority of participants (80%) followed this meal pattern of three main meals. However, data on meal timing in the Brazilian population remain very limited. One of the few available studies, conducted by our group using representative data of Brazilian adults, revealed a wide variation in the timing of meal schedules. The first meal typically occurs between 6:30 h and 9:30 h, while the last meal generally starts around 18:00 h and can extend until nearly 22:00 h [18]. More studies are needed to better understand these patterns and their implications

The aim of the current study was to investigate differences in food intake times between people living in urban and rural areas, as well as between weekdays and weekends, in a representative Brazilian sample. We expected to identify a weekday–weekend pattern with an earlier food intake time in the rural population during weekdays compared to weekends. Additionally, we hypothesized that the eating window would be extended during weekends due to the delayed food intake patterns in both rural and urban residents.

## 2. Materials and Methods

This study included data from the National Dietary Survey (INA), a section of the Family Budget Survey (POF) database specifically focusing on food habits and consumption of the Brazilian population [24]. The survey gathered information about the types and quantities of foods consumed, frequency and timing of food intake, and even cooking methods for certain items like meats and vegetables. It consisted of a nationally representative sample of 34,003 residents over 10 years old [24].

This study utilized public data from the 2008–2009 Household Budget Survey (POF), conducted by the Brazilian Institute of Geography and Statistics (IBGE), which is accessible for consultation online (http://www.ibge.gov.br, accessed on 23 March 2024). The microdata provided by IBGE ensure confidentiality by omitting identifiable information such as household addresses, telephone numbers, and census tract numbers. Brazilian census data are protected by law (Law No. 13,709/2018—General Data Protection Law and Supplementary Law No. 105/2001), ensuring that confidential information is not made available to the public. Ethical principles, such as respect for privacy, confidentiality, and the responsible use of data, are aligned with the guidelines of the Helsinki Declaration and are followed in surveys conducted by IBGE [24,25].

Data collection for the survey occurred over a 12-month period, beginning in May 2008 and concluding in May 2009. Participants were asked to self-report their dietary intake for two non-consecutive days within a seven-day period using a food diary [24]. This was important to minimize memory bias as the recording occurred in real time. Data were collected across all Brazilian states and the Federal District, using weighted sampling to ensure a representative sample [24]. To account for potential deviations from the overall population, the POF sampling weights considered some criteria: family size, selection probability, response rate, geographical and social strata distribution, and other statistical adjustments [25].

The POF (2008–2009) utilized a multi-stage sampling design in which census sector clusters served as the foundation for stratification within each stratum. Stratification was determined by government administrative divisions, urban or rural locale, and income levels as per the 2000 IBGE Census data [24,25]. The adopted criteria to stratify urban and rural areas was based on the division of census sectors from the IBGE Demographic Census, considering geographic and socioeconomic aspects. The areas were classified as urban or rural and subdivided into categories such as capitals, metropolitan regions, and municipalities. Within each geographic stratum, the census sectors were grouped based on the total income of the head of the household, ensuring statistical stratification. This scheme ensured the representation of urban and rural areas in the sample, optimizing the accuracy of the estimates [24,25].

From the initial sample (34,003), only data from adult residents were selected for this study (aged 18–59 years), which excluded 12,983 individuals. To ensure an accurate representation of both weekday and weekend eating habits, we further refined the sample by excluding individuals with missing food diary completion dates (*n* = 398, remaining: 20,622). Additionally, we only included participants who provided two distinct food diary entries, one from a weekday and one from a weekend. This resulted in the exclusion of 14,852 individuals, leaving a final sample size of 5770 participants with complete dietary data for both weekdays and weekends.

For the overall dataset of the survey (POF), the two days were completed randomly, without restrictions on whether they were weekdays or weekend days. However, for this study, only participants who recorded one weekday and one weekend day were selected. Participants with records for two weekdays or two weekend days were excluded from the analysis.

This study focused on the temporal aspects of food intake, which we have termed chrononutritional variables. The specific timing-related variables included First Food Intake Time, Last Food Intake Time, Eating Midpoint, Caloric Midpoint, and Eating Window. We defined First Food Intake Time as any consumption occurring after 05:00 h, and Last Food Intake Time was considered up to 04:00 h. The Eating Midpoint was calculated as the average time between the first and last intake times, using the following formula: Eating Midpoint = (Last Food Intake time—First Food Intake time/2) + First Food Intake time. For example, an individual who consumed their first meal at 08:00 h and their last meal at 20:00 h has a 12 h eating window, resulting in an Eating Midpoint of 14:00 h. The Caloric Midpoint was calculated as the time at which 50% of each individual’s daily calories were consumed. The Eating Window was based on the time between the first and last food intake.

### Statistical Analysis

To characterize the data, we calculated descriptive statistics (proportions and confidence intervals) for sociodemographic data between Brazil’s areas (urban and rural). The chrononutritional variables were described through means and confidence intervals related to the weekday vs. weekend comparison and the urban/rural areas. For categorical variables presented in the sociodemographic proportions, we employed a chi-squared test to assess potential differences in frequencies, whereas a two-way ANOVA was used to explore the influence of the weekday vs. weekend comparison and the urban vs. rural areas on the chrononutritional variables. Following the significant interactions identified by the two-way ANOVA, post hoc comparisons were conducted using the Sidak correction.

Analysis of the time series derived from the two non-consecutive days of food diaries was conducted using Lomb–Scargle periodograms (LSP) to detect potential rhythmic patterns in the chrononutritional variables. The “lomb” R package, specifically the “lsp” function, was employed for computing Lomb–Scargle periodograms, chosen for its suitability in handling time series with irregular sampling intervals [26]. This method is widely utilized in chronobiology due to its robustness in addressing unevenly spaced time series, along with its ability to provide statistical significance levels for each peak identified in the periodogram [26,27]. The irregularity in our samples’ time series stems from incomplete data, attributed to sporadic missing observations occurring throughout the data collection period spanning 2008 to 2009.

Both descriptive and inferential analyses were adjusted for the samples’ complex design, which accounted for the potential clustering of participants within specific groups. This involved utilizing either the “svy” commands in Stata 16 or the “survey” package in R 4.2.1 throughout the data analysis [28]. A significance level of *p* < 0.05 was adopted for all analyses.

## 3. Results

The sociodemographic variables are described in Table 1 comprising the total sample (*n* = 5770) and stratified by the areas (urban and rural). This study consisted of 76.2% of people living in urban areas, whereas 23.8% lived in rural areas. Most of the frequencies of the sociodemographic variables differed between urban and rural areas. There was a slight predominance of females in urban areas (50.5% vs. 49.5% of males); the opposite pattern was observed in the rural areas comprising a male predominance (54.4% vs. 45.6% of females). In addition, people reporting 0–10 years of education were in a slight majority in the urban areas (51.3% vs. 48.7% reporting more than 11 years of education). On the other hand, the frequency of people reporting 0–10 years of education was higher (80.6%) than people reporting more than 11 years of education (19.4%) in the rural areas. Most people declared themselves white (51.1%) in the urban areas followed by black/brown declarations (47.9%). An opposite pattern was observed in the rural areas with most people declared black/brown (67.1%) followed by white (31.3%).

The comparison between weekdays and weekends revealed significant main effects in three chrononutritional variables (Table 2). The first food intake time occurred earlier on the weekdays (07:42 h) compared to weekends (07:53 h; *p* < 0.001). However, the last food intake time showed an opposite pattern occurring later in the weekdays (20:07 h) compared to the weekends (19:59 h; *p* = 0.04). Although these differences seem small, they result in a significant difference in the length of the eating window with an increased duration during the weekdays (12.42 h) compared to the weekends (12.09 h, *p* < 0.001). On the other hand, both the eating and caloric midpoints did not vary between the weekdays and the weekends.

The urban/rural area where the participants lived influenced the food intake time on most of the chrononutritional variables. In urban areas the first food intake time occurred later than in the rural areas (07:57 h vs. 07:17 h respectively; *p* < 0.001), while the last food intake time followed the same pattern, occurring later in urban areas compared to rural areas (20:12 h vs. 19:39 h respectively; *p* < 0.001) regardless of the day of the week. These outcomes led to differences in the eating and caloric midpoints between urban and rural areas. Both of these chrononutritional variables occurred later in the urban areas than in the rural areas (urban: 14:04 h and 13:36 h vs. rural: 13:28 h and 12:59 h; *p* < 0.001 in both). No statistical differences in the chrononutritional variables were observed in the interactions of the weekday vs. weekend comparison x urban/rural area.

**Figure 1:**
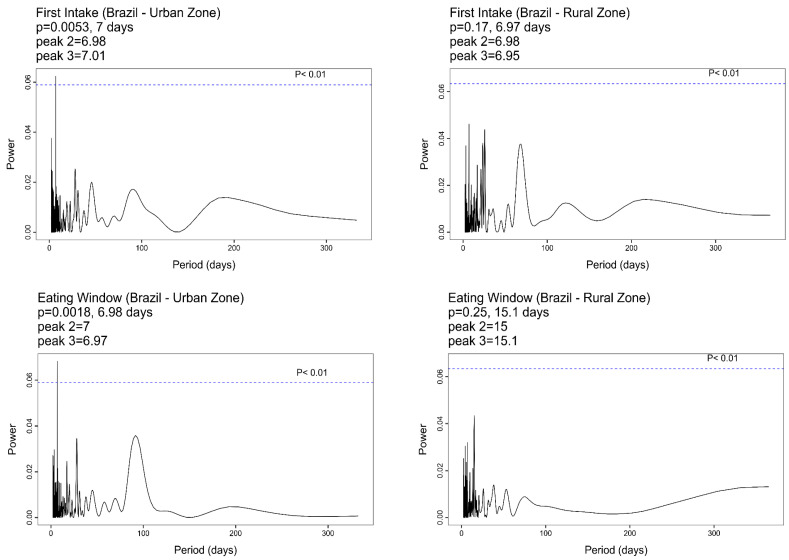
Lomb–Scargle periodograms of the first food intake time (two figures **above**) and the eating window (two figures **below**) by area (urban and rural, respectively). The first peak represents the highest power value described by the p-value followed by the period in days. The second and third peaks have lower power. The period is described in days.

Analysis of the time series (365 days) considering the areas separately (urban and rural) detected a weekly rhythmic pattern in both the time of first food intake (7 days) and the eating window (6.98 days) only in the urban area (Figure 1). The other chrononutritional variables (last food intake time, eating midpoint, and caloric midpoint) did not show significant peaks in their periodograms in either of the areas (Supplementary Material, Figures S1–S3).

## 4. Discussion

This is the first report to identify a weekday–weekend rhythmic pattern in food intake timing in both urban and rural populations, and to examine differences in these patterns between weekdays and weekends. Firstly, our study demonstrates a weekly rhythm exclusively in urban areas. There was no statistically significant weekly rhythm in food intake times in rural residents, which allows us to reject the hypothesis initially designed for this study. It suggests the absence of a strong social determinant distinguishing weekends from workdays. Secondly, when comparing urban and rural areas, we found that individuals residing in urban areas had significantly later first food intake times compared to those living in rural areas. The same pattern was observed for the last food intake, with urban dwellers also having later last food intake times. Thus, urban residents had both later eating and caloric midpoints. Thirdly, our results indicate weekday–weekend differences in the temporal aspects of food intake as a main effect. We found that the first food intake times during weekdays were significantly earlier than on weekends, regardless of the areas where the participants lived, which corroborates our initial hypothesis. By contrast, the last food intake times were later during weekdays than on weekends. These findings reject our hypothesis of a longer eating window on weekends due to the finding of a longer eating window during the weekdays in our study, which was similar to a recent Chinese cross-sectional study [29].

Two studies conducted by our group, using the same database as this study (POF 2008–2009), have recently been published. These studies show regional variations in food intake times among Brazilians and how these are associated with BMI and obesity [18,30]. Crispim et al. demonstrated a significant effect of food intake times on the risk of being overweight and obese [18]. Additionally, using logistic regression analysis, we found that later timing of the first and last food intake, as well as higher calorie consumption after 21:00 h, was associated with an increased risk of overweight and obesity in the Brazilian population. Another study that investigated seasonal aspects in chrononutritional variables found a positive effect of latitude on the eating midpoint, and first and last food intake times [30]. The delayed food intake time suggested that the use of nutritional planning based on intervention policies on healthier eating times would be essential for the population located at high latitudes in Brazil [30]. This chrononutritional planning advances eating times, which could help to prevent overweight and obesity in the Brazilian population.

Some studies have investigated differences in food consumption between weekdays and weekends demonstrating both increased eating episodes and preference for snacks during the weekdays compared to weekends [31,32,33,34]. Moreover, eating either during the daytime or in the evening was reduced on weekends [35]. However, data from NHANES (2003–2012) have found the opposite effect, showing increased total energy intake and a less healthy diet during weekends compared to weekdays. This highlights the role of fast foods in contributing to the high-calorie intake and poorer diet on weekends [21]. Considering our results, we can infer that these differences are specific to urban areas, as no weekly pattern was observed among rural dwellers, suggesting they tend to maintain the same eating times on both weekends and weekdays.

The longer working hours and commutes tend to delay sleep and the food intake times routine in people living in urbanized cities [36,37,38], leading to later first and last food intake times, as well as the later eating and caloric midpoints found in our study. Breakfast skipping is a common practice in urbanized cities [19,39], thus individuals delay their first food intake time and swap this high-quality meal for ultra-processed snacks [40,41]. Furthermore, the increased opportunity for social activities in urban areas gives access to several food options (e.g., food delivery services and convenience stores) after an extended work journey or social event; this scenario thus contributes to activities late into the night, delaying the last food intake times [1,10]. This phenomenon usually develops into a chronic circadian rhythm delay, not just affecting food intake times but also disrupting other circadian-oriented behaviors such as the sleep/wake cycle [14,42].

People living in urban areas have societal routines/pressures mentioned above as well as other specific conditions related to circadian biology. Reduced exposure to natural daylight in work/school settings alters circadian rhythms influencing food intake times across the days. A laboratory study conducted under constant routine conditions demonstrated that meal times synchronize peripheral oscillators [36], which helps explain the recognized correlation between the circadian system and food intake. Thus, the 7-day rhythmic pattern detected in this study suggests that individuals living in urban areas exhibit weekly variations in food intake times that align with their other weekly oriented circadian rhythms, such as the sleep/wake cycle. This relationship suggests that the weekly irregularity of food intake times may be a pivotal factor contributing to the social jetlag in the weekly sleep/wake cycle [43,44]. Urban residents are thus prone to experience delayed food intake times and a longer eating window during weekdays, which drives both health and well-being impairments due to circadian misalignment [8,45]. On the other hand, exposure to natural daylight in people living in rural areas is reflected in both regular food intake times and sleep/wake times during both weekdays and weekends [46].

By contrast, a few studies conducted in both low-income and high-income countries have reported increased risk for overweight/obesity in rural populations [47,48]. The authors have suggested that preservation food methods (e.g., salting and smoking) when there is restricted electricity might be an explanation for these findings [19,49]. Moreover, a population-based study conducted by Trivedi and colleagues in the USA (NHANES 1999–2006) identified poor meal habits in rural populations, which were characterized by a higher consumption of sugary beverages and lower intakes of fiber and fruits, highlighting a higher propensity for obesity in rural than in urban areas [47].

The forces influencing work and school routines in urbanized societies have been related to consistent food consumption patterns during the week [19]. A pivotal population-based study undertaken in Brazil using the same sample as this study (POF 2008–2009) found that daily energy intake was higher during weekends [33]. In addition, carbohydrates were the main components of this energy intake during weekdays, in contrast to the total saturated and trans fats that were identified more frequently during weekends [33]. During the weekends (when the eating window was shorter), the total energy intake was higher than on weekdays due to social activities that could change food intake favoring the consumption of fast foods and ultra-processed foods leading to low-quality food consumption [33]. Through the lens of chrononutrition, it is possible to visualize a potential factor for overweight and obesity in the Brazilian population [33], namely, the reduced eating window observed on weekends in our study. This occurred at the same time as the higher calorie intake identified during this period [33]. This situation highlights a concern about the weekly variation in eating behavior and the potential role of social activities in driving these irregular meal time patterns.

Despite the lack of studies demonstrating a variation in meal times during the week, results with data from one of the most important national population-based surveys were published recently [50]. Evidence from the National Health and Nutrition Examination Survey (NHANES 2017–2018, USA) corroborates our findings showing an early first food intake time and a late last food intake time during weekdays of the whole sample [50]. In addition, these findings revealed a longer eating window during weekdays similar to our study. However, the study did not analyze the weekly rhythmic pattern of urban and rural samples, nor did it compare them.

Previous studies have shown that the influence of the workplace during the weekdays and social obligations during weekends may be responsible for the common pattern of more meals and snacks during the weekdays compared to weekends [21,33,51]. There is a survey that recorded the main meals in the evening relative to weekdays in the US population [21] that highlighted the potential effect of work, study, and other obligations [37,38] to spread meal times over a longer period during weekdays. In addition, the length of the commute is a critical factor that affects meal times in populations residing in urban cities [37] and contributes to an extended eating window during weekdays. Conversely, the free time available during the nonwork days on weekends allows food intake at more favorable times and includes less breakfast skipping and less ingestion of multiple snacks throughout the day [21].

This study provides a comprehensive analysis of cross-sectional food diary data from a large population-based sample in Brazil. A key strength of this research lies in its ability to elucidate weekday–weekend variations in food intake timing and to identify disparities in temporal eating patterns between urban and rural populations. Such insights are helpful for the development and implementation of effective public health nutrition policies that are tailored to the specific needs and behaviors of diverse demographic groups. Thus, chrononutrition (the time of eating) emerges as a crucial factor for healthcare professionals to consider when promoting healthy eating habits. By recognizing the influence of both the day of the week and the geographic location on dietary patterns, practitioners can design more targeted and impactful interventions.

This study has some limitations. This is a cross-sectional study, and while we conducted analyses to adjust for potential confounding factors, this study design inherently limits the ability to establish causal relationships, with reverse causality being a concern. The instrument used to gather information on food consumption, although validated and widely used in scientific studies, is subjective and relies on the motivation of the interviewed participants. In addition, the reduced sample size may have affected the statistical analyses, although it was essential to ensure an accurate representation of both weekday–weekend eating habits including only individuals with complete food diary data (details in the Methods Section). Reduction in the sample size in complex sampling designs may increase the chance of producing bias due to not accurately representing the population distribution within each stratum [52,53]. Even with the relevant reduction in sample size, it remained satisfactory to perform analyses with participants from all regions of the country, albeit not evenly distributed. The sample size of 5770 individuals remained sufficiently large; however, future studies on larger groups are needed. Although the data were from over 10 years ago, they remain one of the few databases that still provide a comprehensive and representative overview of eating times in the Brazilian adult population. Despite potential changes in eating habits following the COVID-19 pandemic, these data are crucial for understanding long-term trends and helping to formulate hypotheses to be tested in the future.

## 5. Conclusions

Our study demonstrates that urbanization contributes to delayed food intake times and the maintenance of a rhythmic 7-day pattern, which was not observed in rural areas. These findings help to explain why people living in urban environments face the negative consequences of unhealthy eating schedules, such as being overweight, with an increased risk of obesity, and circadian misalignment. Additionally, the results highlight that during weekdays, the first food intake occurred significantly earlier and the last food intake occurred significantly later than on weekends, leading to a longer eating window. These insights will be valuable for healthcare professionals and policymakers in promoting healthier food intake schedules, taking into account both the days of the week (weekdays and weekends) and the participant’s place of residence (urban or rural).

## Figures and Tables

**Table 1:** Sociodemographic characteristics of the sample according to the areas.

		Areas	
	Brazil (*n* = 5770)	Urban (*n* = 4400)	Rural (*n* = 1370)
Sociodemographic Variables	% (95% CI)	% (95% CI)	% (95% CI)
Sex			
Male	50.4	49.5 *	54.4 *
	(49.0–51.7)	**(47.9–51.1)**	**(52.0–56.8)**
Female	49.6	50.5 *	45.6 *
	(48.2–51.0)	**(48.9–52.1)**	**(43.2–48.0)**
Age (years)			
18–25	21.3	21.1	22.4
	(19.8–22.9)	(19.3–22.9)	(19.3–25.5)
26–35	28.1	28.3	27.2
	(26.2–30.0)	(26.1–30.4)	(23.7–30.7)
36–45	26.1	26.1	25.9
	(24.3–27.8)	(24.1–28.1)	(22.6–29.3)
45–59	24.5	24.5	24.5
	(22.8–26.2)	(22.6–26.4)	(21.2–27.7)
Years of education			
0–10	56.6	51.3 *	80.6 *
	(54.4–56.7)	**(48.8–53.7)**	**(77.8–83.5)**
>11	43.4	48.7 *	19.4 *
	(41.3–45.6)	**(46.2–51.2)**	**(16.5–22.2)**
Race/ethnicity			
White	47.5	51.1 *	31.3 *
	(45.3–49.6)	**(48.5–53.6)**	**(27.1–35.4)**
Black/Brown	51.5	47.9 *	67.1 *
	(49.2–53.5)	**(45.4–50.5)**	**(62.8–71.5)**
Asian/Indigenous	0.7	0.6 *	1.3 *
	(0.4–1.1)	**(0.3–1.0)**	**(0.2–2.4)**
Do not know	0.3	0.4 *	0.3 *
	(0.1–0.6)	**(0.1–0.6)**	**(0.0–0.7)**
BMI (kg/m^2^)			
<24.9	54.4	53.4 *	58.7 *
	(52.5–56.3)	**(51.2–55.6)**	**(55.4–62.0)**
25–29.9	32.2	32.9 *	29.3 *
	(30.5–33.9)	**(30.9–34.8)**	**(26.2–32.3)**
≥30	13.4	13.7 *	12.0 *
	(12.1–14.7)	**(12.2–15.2)**	**(9.4–14.6)**

National Household Budget Survey (POF/IBGE 2008–2009). * Chi-squared test: *p* < 0.05 in bold. Data are indicated by the frequency (%) and the confidence interval (95% CI).

**Table 2:** Isolated and interaction effects according to the weekday vs. weekend and area comparison related to the chrononutritional variables.

	Weekday vs. Weekend		Area		Effects/Interaction		
	Weekday	Weekend	Urban	Rural	Weekday vs. Weekend	Area	Weekday vs. Weekend × Area
Chrononutritional Variables	Mean (95% CI)	Mean (95% CI)	Mean (95% CI)	Mean (95% CI)	F (*p*-Value)	F (*p*-Value)	F (*p*-Value)
First Food Intake Time (h:min)	**07:42**	**07:53**	**07:57**	**07:17**	36.04	64.43	0.38
	**(07:37–07:46)**	**(07:48–07:58)**	**(07:51–08:03)**	**(07:10–07:25)**	(<0.001 *)	(<0.001 *)	(0.54)
Last Food Intake Time (h:min)	**20:07**	**19:59**	**20:12**	**19:39**	4.11	36.03	3.55
	**(20:02–20:12)**	**(19:53–20:04)**	**(20:05–20:18)**	**(19:30–19:47)**	(0.04 *)	(<0.001 *)	(0.06)
Eating Midpoint (h:min)	13:54	13:56	**14:04**	**13:28**	2.72	87.64	3.17
	(13:51–13:58)	(13:52–13:59)	**(14:00–14:09)**	**(13:22–13:34)**	(0.09)	(<0.001 *)	(0.07)
Caloric Midpoint (h:min)	13:29	13:21	**13:36**	**12:59**	3.77	31.77	0.01
	(13:22–13:35)	(13:15–13:28)	**(13:29–13:43)**	**(12:48–13:10)**	(0.05)	(<0.001 *)	(0.96)
Eating Window (decimal hour)	**12.42**	**12.09**	12.25	12.30	30.05	0.86	1.66
	**(12.32–12.53)**	**(11.96–12.23)**	(12.12–12.38)	(12.15–12.45)	(<0.001 *)	(0.35)	(0.20)

Two-way ANOVA (factors: weekday vs. weekend; urban vs. rural). * Statistical significance (*p* < 0.05) in bold.

## Data Availability

Available upon request to the corresponding author.

## Supplementary Materials

The following supporting information can be downloaded at: https://www.mdpi.com/article/10.3390/nu17010108/s1, Figure S1: Lomb–Scargle periodograms from the last food intake time by the geographic areas (urban and rural zones); Figure S2: Lomb–Scargle periodograms from the eating midpoint by the geographic areas (urban and rural zones); Figure S3: Lomb–Scargle periodograms from the caloric midpoint by the geographic areas (urban and rural zones).
