# Supplementary material for: Weekday–Weekend Differences in Chrononutritional Variables Depend on Urban or Rural Living
Source: Nutrients. 2024 Dec 30;17(1):108. doi: 10.3390/nu17010108 (PMC11723074; doi:10.3390/nu17010108)
Supplement: Supplementary file 1 [file nutrients-17-00108-s001.zip › nutrients-3360542-supplementary.docx]

**Supplementary Material**


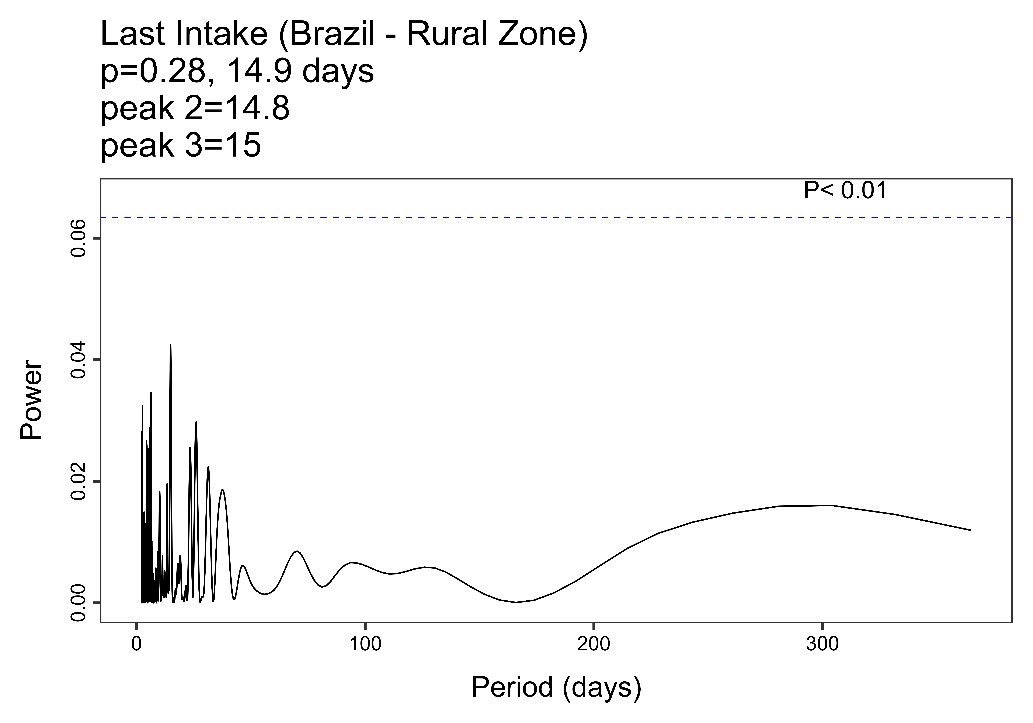

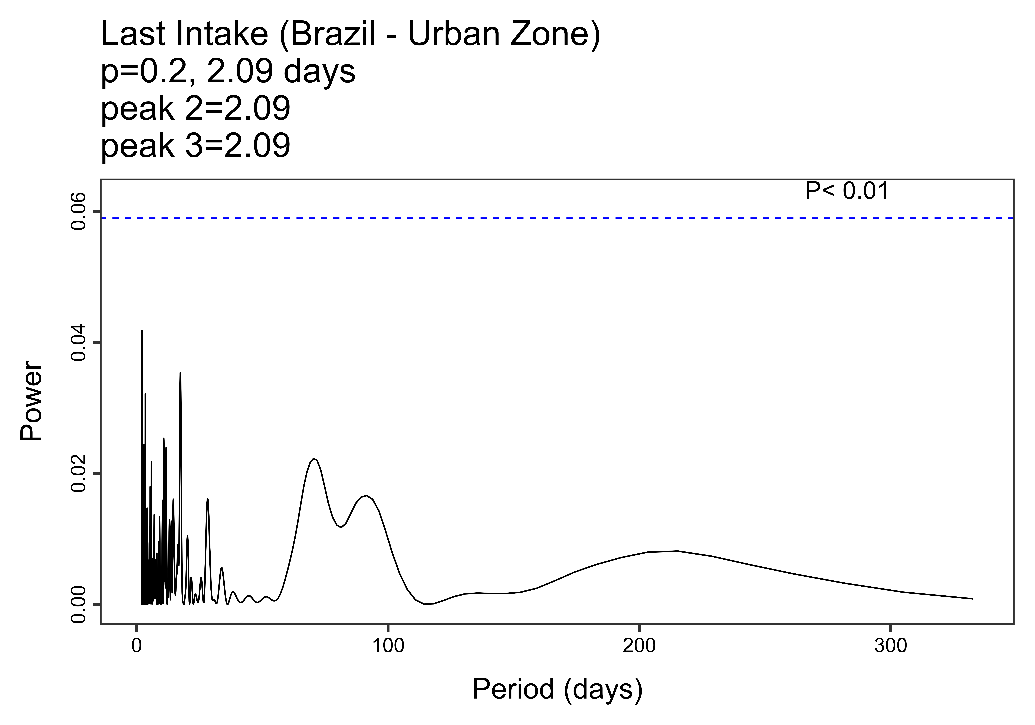


Figure S1 - Lomb-Scargle periodograms of the last food intake time by area (urban and rural, respectively). The first peak represents the

highest power value described by the p-value followed by the period in days. The second and third

peaks have lower power. The period is described in days.


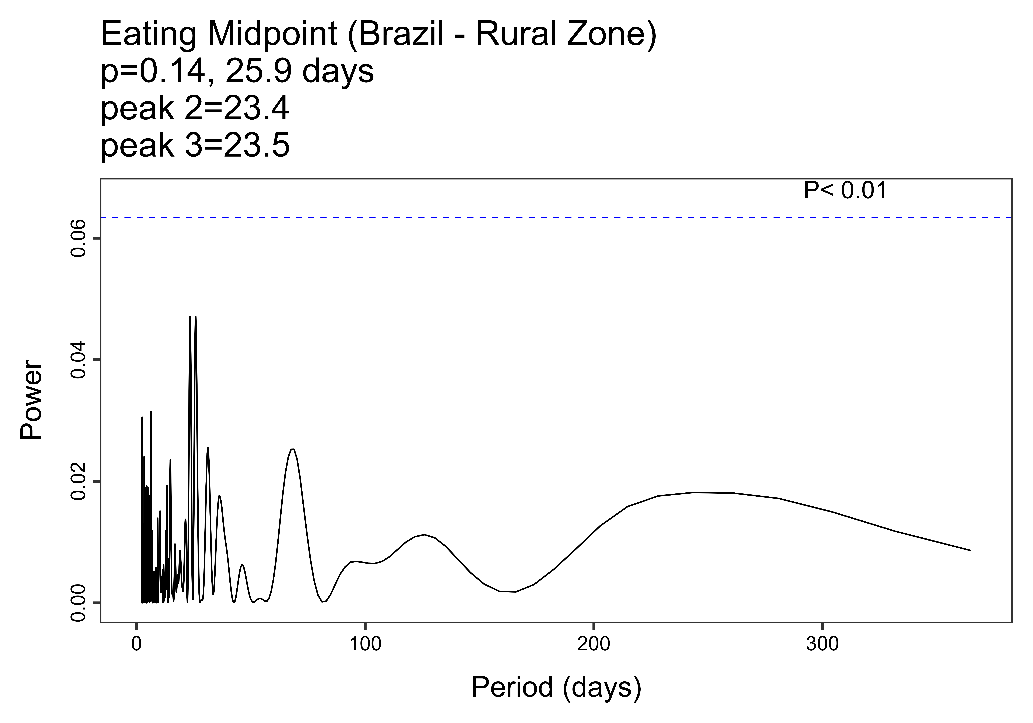

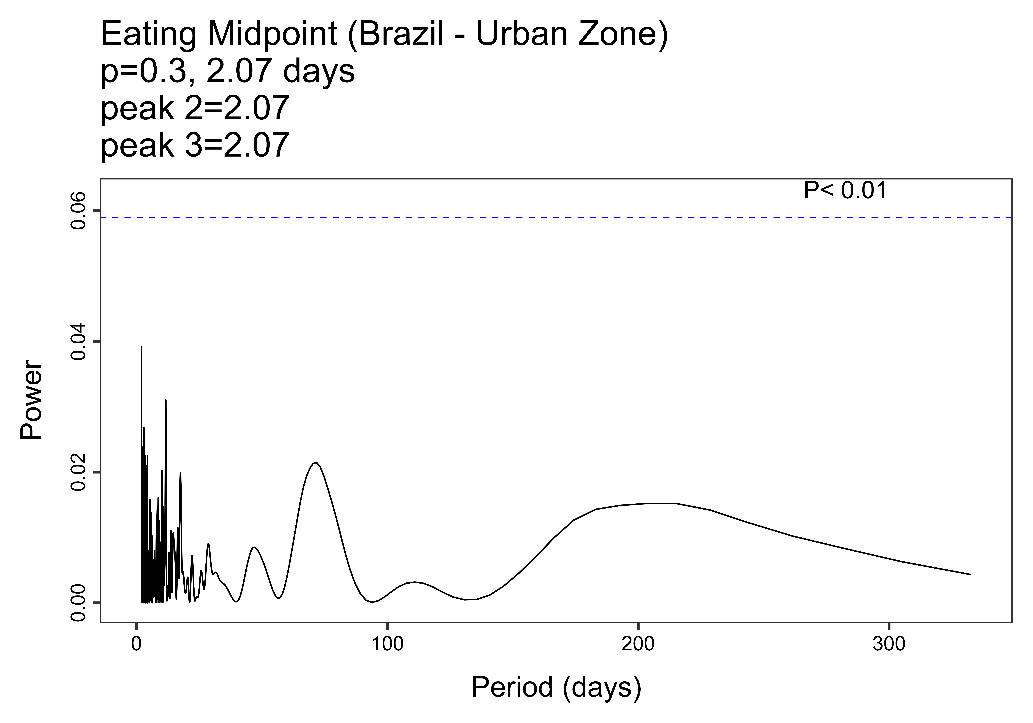


Figure S2 - Lomb-Scargle periodograms of the eating midpoint by area (urban and rural, respectively). The first peak represents the

highest power value described by the p-value followed by the period in days. The second and third

peaks have lower power. The period is described in days.


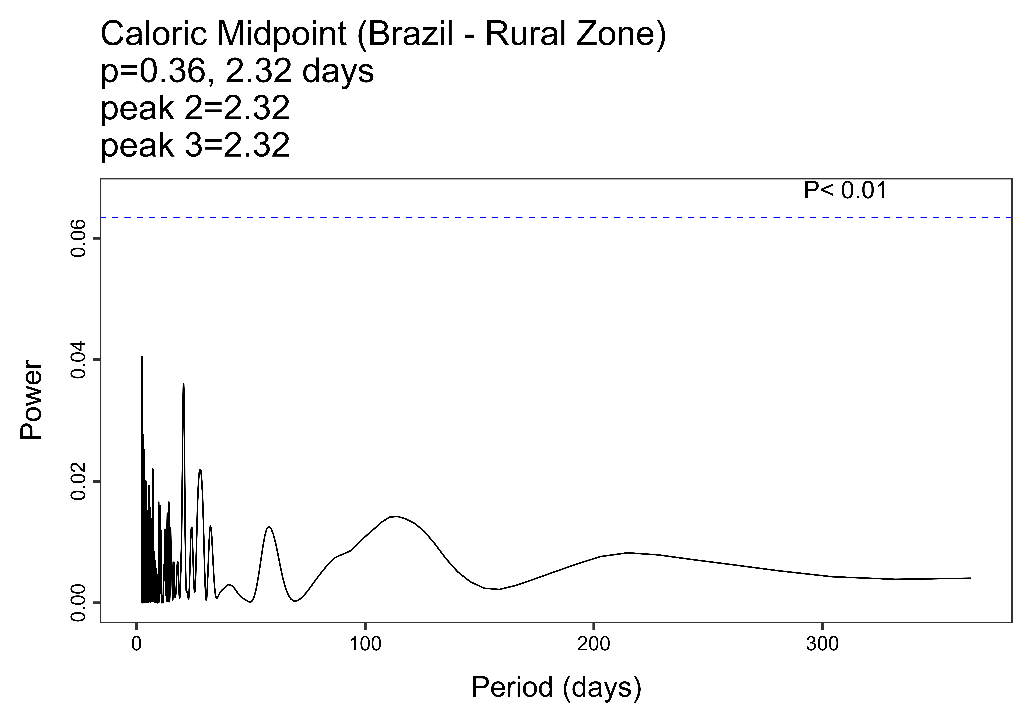

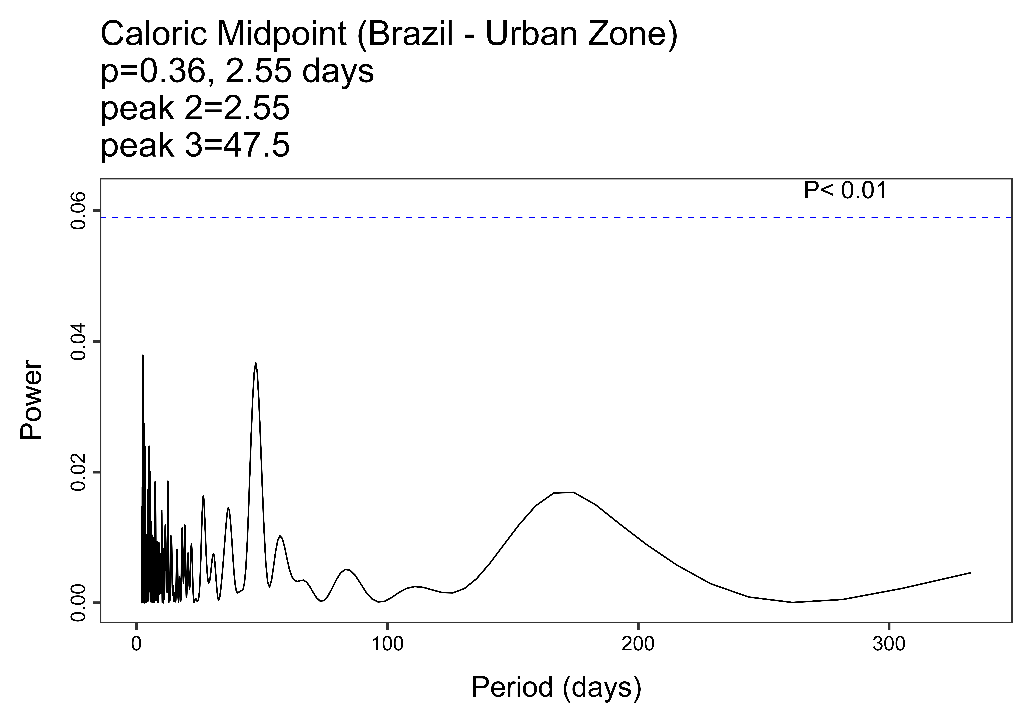


Figure S3 - Lomb-Scargle periodograms of the caloric midpoint by area (urban and rural, respectively). The first peak represents the

highest power value described by the p-value followed by the period in days. The second and third

peaks have lower power. The period is described in days.
